# Evaluation of Staffordshire, Stoke on Trent Allied Health Professionals preceptorship programmes: a mixed method UK study

**DOI:** 10.1186/s12909-023-04515-7

**Published:** 2023-08-21

**Authors:** E. Salt, K. Jackman, A. V. O’Brien

**Affiliations:** 1 University Hospitals of Derby and Burton NHS Foundation Trust, Burton-On-Trent, UK; 2 School of Allied Health Professions, Keele University, Newcastle, UK; 3 Staffordshire and Stoke On Trent (SSOT) Allied Health Professionals (AHP), Staffordshire, UK; 4 Midlands Partnership Foundation Trust, Staffordshire, UK

**Keywords:** Preceptorship, Allied health professions, Staffordshire

## Abstract

**Aim:**

This study aimed to evaluate current preceptorship provision across AHP professions in the Staffordshire, Stoke on Trent (SSOT) region of England to improve consistency, share and optimise best practice.

**Background:**

Preceptorship, defined as a period of structured transition from newly qualified to an independent practitioner, is thought to improve recruitment and retention of staff and ultimately improve patient care. During the COVID pandemic, SSOT recognised the potential for graduates to lack confidence having had reduced clinical exposure as pre-registration students, and so a likely increased need to support newly qualified staff, and to evaluate existing AHP preceptorship provision.

**Methods:**

An explanatory sequential mixed methods design, utilising a cross sectional survey questionnaire and two subsequent focus groups, explored existing AHP preceptorship in SSOT in 2021.

Following ethical approval respondents were recruited via professional networks who completed an online survey questionnaire. Two subsequent focus groups enabled an in-depth exploration of survey results. Descriptive statistics summarised survey data and thematic analysis was used to describe focus group findings.

**Results:**

SSOT AHPs (*n* = 217; 26% preceptees; 47% preceptors) participated in the survey questionnaire and 17 AHPs in the focus groups. 57% of preceptees rated existing preceptorship programmes to be “somewhat, or not effective”. Preceptors reported feeling unprepared for their role. Both preceptees and preceptors reported that, post pandemic, most existing programs required revisions to be fit for purpose. Ten pragmatic summary recommendations were made.

**Conclusions:**

Allied Health Professions Preceptorship in SSOT was found to be inconsistent, poorly understood and inadequate. Revisions to preceptorship programs across Staffordshire and Stoke on Trent NHS Trusts have been instigated to reflect changes in AHP practice since the COVID pandemic.

**Supplementary Information:**

The online version contains supplementary material available at 10.1186/s12909-023-04515-7.

## Introduction

One ambition in the long-term plan for the National Health Service (NHS) in England, is to make the NHS a better place to work [1]. In the Midlands of England, the Staffordshire, Stoke on Trent (SSOT) Allied Health Professionals (AHP) Faculty identified that Preceptorship was a priority to help achieve this ambition for AHPs. A preceptorship has been defined as a period of structured transition for a newly registered practitioner (the preceptee), during which they will be supported by a supervisor (the preceptor).

Preceptorship was recognised by England’s Department of Health [2] regarding modernising careers in 2006. More recently a nursing, midwifery and AHP implementation framework for preceptorship programs was published based on extensive stakeholder engagement in conjunction with reviewing preceptorship models and approaches worldwide [3]. Later, Health Education England published national Preceptorship standards [4]. Whilst it is acknowledged that preceptorship should be rolled out as part of the UKs national strategy to modernisation of health care, preceptorship is currently a recommendation rather than a mandated requirement in England.

Preceptorship is intended to develop confidence as an autonomous professional, refine skills, values and behaviours, and assist new graduates to continue their journey of life-long learning [5, 6]. It has been reported that by improving newly qualified practitioners' knowledge reduces errors and improves health care provision [5]. Published preceptorship programmes have been based around the four pillars of learning: clinical practice (developing knowledge, skills and behaviours to support safe, patient centred care), facilitated learning (skills for learning in the workplace and supporting others to learn), leadership (learn about leadership styles and skills including inclusivity), and evidence and research (ability to use evidence from a range of resources including service user involvement) [7]. Potentially, post COVID, preceptorship is now more important than ever.

Allied Health Professionals are comprised of 14 different professional disciplines and are the third largest workforce in England’s’ National Health Service, with over 170,000 AHP practitioners registered [8]. England's National Health Service is divided geographically into 42 Integrated Care Systems (ICS). ICSs are partnerships that bring together health providers, local authorities and others to improve health and reduce inequalities. Staffordshire and Stoke ICS support 1.1 million people and consist of four NHS providers (two acute hospital Trusts, once community Trust and one mental health Trust) along with an ambulance service. It covers all aspects of health and social care. Data obtained (to the nearest whole time equivalent) from three out of the four Trusts (at the end of June 2021), suggests that SSOT Integrated Care System (ICS) has approximately 1,500 AHPs. There was a belief within the SSOT ICS that Preceptorship was variable, with some Trusts providing in-house preceptorship programmes, others providing on-line purchased programmes and others not providing any preceptorship. For the Trusts providing preceptorship programmes it was unclear if they were fulfilling AHP preceptee needs. In addition, it was of interest to understand how the preceptorship programmes were being delivered e.g., whether they were profession specific or generic.

### Rationale for ‘preceptorship’ study

Initial investigations established that little was known about the provision of AHP preceptorship in SSOT. Mindful of the revised and unique needs of 2021 graduates, whose student experience had been significantly altered due to the COVID pandemic, concern was expressed about potential increased risk of newly qualified AHP attrition. Consequently, the SSOT AHP Council commissioned the AHP Faculty to review and appraise existing preceptorship provision with a view to enhancing newly qualified AHP recruitment and retention. Health Education England (HEE) AHP Faculty funding facilitated progression of the project and the recruitment of a dedicated project manager.

#### Project aims

Three key aims were established for the study:

1. To identify the current provision of AHP preceptorships across SSOT region.
2. To understand the impact of the COVID-19 pandemic on newly qualified AHPs and whether the current preceptorships were fit for purpose.
3. To make recommendations to improve SSOT AHP preceptorship programmes.

## Methods

A mixed methods explanatory sequential design optimising both quantitative and qualitative approaches was selected to achieve the study aims. The assumption was that the quantitative data from a survey would inform the planning of the qualitative phase (the focus groups) of the study.

Creswell describes mixed methods as gathering quantitative (close-ended) and qualitative (open-ended) data, integrating the two, and drawing interpretations based on the combined data sets’ [9, 10]. This method allows for mixing of both paradigms of deduction (which explores a hypothesis by testing it- an approach frequently used in a quantitative research) and induction (which involves open-ended observations to logically explain findings; an approach frequently used in a qualitative research) in a holistic way [11]. By using a mixed methods approach, it was hoped priority improvements and recommendations could quickly be identified to inform changes to improve AHP preceptorship programmes in Staffordshire, Stoke on Trent.

A sequential mixed method study design was used:

1. An online self-completed cross-sectional survey questionnaire (via Microsoft forms) aimed at qualified AHPs working within the SSOT ICS.
2. Consenting survey respondents were recruited to participate in two subsequent focus groups with current SSOT newly qualified AHPs (preceptees) and experienced AHP preceptors/ managers (preceptors).

In this design, survey responses informed the subsequent interview topic guide used for the focus groups, which enabled greater understanding of preceptor and preceptee experiences in SSOT. For example, barriers to participation in preceptorship programmes and participants’ recommendations for improvements for newly qualified AHP staff could be explored in more detail.

It has been reported that inconsistencies, uncovered using a mixed method approach, strengthen research by providing information that would not necessarily be discovered using a single method approach [12]. Consideration was given to how contradictory views would be managed to ensure transparency of reporting [10, 13]. The McGill Mixed methods Appraisal Tool (MMAT) guided the reporting of this research.

The project team included researchers with a background in qualitative research (AOB),and a background in quantitative methods (ES), thus, strengthening the overall quality of the study.

### Quantitative component

The survey was designed by a sub-group of the SSOT AHP Faculty Committee and piloted by five newly qualified AHPs/ ‘critical AHP friends’ outside SSOT ICS across England’s West Midlands region. Following the pilot, minor amendments were made to clarify questions. A Microsoft form was selected as the online platform supported across all healthcare providers within the ICS, to optimise responses.

The survey included 65 questions in six sections: demographic and profession related details; (including where the AHP was currently practicing, banding of post and whether they were a newly qualified); details of preceptorship programs and supervisory support, questions relating to delivery, usefulness, and finally AHP personal experiences of preceptorship.

#### Sample

##### Inclusion criteria:

The eligibility criteria for survey respondents to be included in the analysis were:

i) Self–reported as qualified Health and Care Professionals Council (HCPC) registered Allied Health Professionals [1].
ii) Employed in the Integrated Care System of Staffordshire and Stoke on Trent

##### Exclusion criteria:

i Support worker/ un-qualified health professional assistant
ii Registered AHP working outside of SSOT ICS

#### Sample size

No organisation was able to provide a complete inventory of AHP employees by grade or discipline, but estimates were identified for the proportion of AHPs in the four NHS providers in 2021 (Table 1).

**Table 1:** Estimated 2021 AHP numbers in Staffordshire and Stoke on Trent ICS

(NHS) Employer provider organisation	AHP/ total workforce (*n* = 25,400)	% of total workforce
1	957/8,500	11
2	951/4,500	21
3	450/11,000	4.1
4	60/1,400	4.2
PIVO^a^	Unknown	Unknown
**TOTALS in survey**	**2418/25,400**	**15.6**

Key: *PIVO*^a^ Private independent and voluntary organisations

Given the exploratory nature of the survey a formal sample size was not calculated. No current data exists to accurately identify the number of AHPs working in Stafford and Stoke on Trent ICS.

#### Data analysis

An exploratory analysis used descriptive data, expressing means, SD and percentages where appropriate. Anticipated limited sample size prohibited any further detailed statistical analysis.

#### Recruitment

The project team used a snowball method of recruitment using social media, emails and AHP networks, and NHS Trust communications (briefings and screen savers). Every effort was made to recruit AHPs working in the private, independent, and voluntary organisational sectors. The survey closed on 24/05/21 after 19 days due to the short timeframe of the project manager contract. This was an extension of the original 14 days as it included a bank holiday. The response rate was monitored throughout. The initial response from newly qualified AHPs was disappointing, so additional reminders were sent on Microsoft chat, as well as daily posts on social media. Trusts that had poor response rates were targeted specifically via email and via AHP Faculty members to enhance response rate. Survey data were analysed in Excel version 2016 and summarised using descriptive statistics.

Overall, the survey took respondents approximately 20 min to complete. Preceptee survey responses (*n* = 60) were often incomplete, but it is not known if this was due to a lack of knowledge about preceptorship, a lack of understanding about the question, or just a reluctance to comment. An element of survey fatigue was also possible as a greater percentage of unanswered questions were in the last third of the questionnaire. Most answer options were multiple choices, with subsequent branching to increase speed of completion. There were 21 free text answer opportunities given to allow respondents to clarify their responses e.g., listing adaptations made due to COVID and requesting results and/ or participation in focus groups.

### Qualitative component

Two online focus groups were scheduled to optimise participation, with minimal disruption to clinical services. The research team were mindful that during the COVID-19 pandemic most AHPs were familiar with virtual discussions via Microsoft Teams platform. Consequently, this format was chosen, with additional benefit of ensuring compliance with pandemic social distancing measures and convenience; enabling busy clinicians, across the four main NHS provider organisations maximum opportunity to participate.

#### Sample & recruitment

Survey participants who had had expressed an interest in being involved in a focus group were sent the FG Participant Information Sheet and a consent form. On successful completion and email return, participants were sent the calendar invitation link to participate in the relevant MS Teams focus group.

#### Sample size

The focus groups aimed to recruit between 6–10 participants. This figure has been recommended to provide sufficient meaningful open discussion in a group setting [14].

#### Data analysis

MS stream audio recordings from both FGs were revised and analysed alongside verbatim transcripts by the research team (AOB, ES, KJ). Thematic analysis, which is less constrained by theoretical frameworks, was selected as the appropriate qualitative tool for relatively uncomplex analysis [15]. All three authors independently identified semantic and latent codes before they met to debate, refine and amalgamate codes. Sub themes and subsequently final themes were agreed and identified as per [15] thematic analysis approach. Any discrepancies were resolved through consensus to enhance trustworthiness. This process was conducted first for the preceptee FG and then again (separately) for the Preceptor FG.

#### Focus group preparation

Each FG aimed to further explore and gain additional insights relating to the survey results. The pre-determined topic guide was used to facilitate the discussion and included open questions, with follow up prompts as necessary. Prior to each FG the lead facilitator (ES or AOB) and observer (KJ) independently documented their preconceptions and expected outcome of the discussions, to proactively identify project team assumptions and limit potential bias.

#### Procedure

Each group was led by an experienced facilitator and observed by an independent observer who made additional notes. Consent was reiterated at the start of each FG, which was audio-recorded on MS Teams. To promote honest candid responses, participants were reminded that anonymity would be guaranteed, and any quotes subsequently used would be non-identifiable to an individual or their employing organisation.

The first FG for preceptees (newly qualified AHPs) was held on 23.06.21.; the second for preceptors (experienced clinicians/ managers) on 24.06.21. Prior to the second FG the project team met to share provisional thoughts on how discussions from the preceptee focus group should influence the second discussion. Initial key discussion topics identified in the preceptee group were subsequently further explored with the preceptors.

### Data integration

**Fig. 1:**
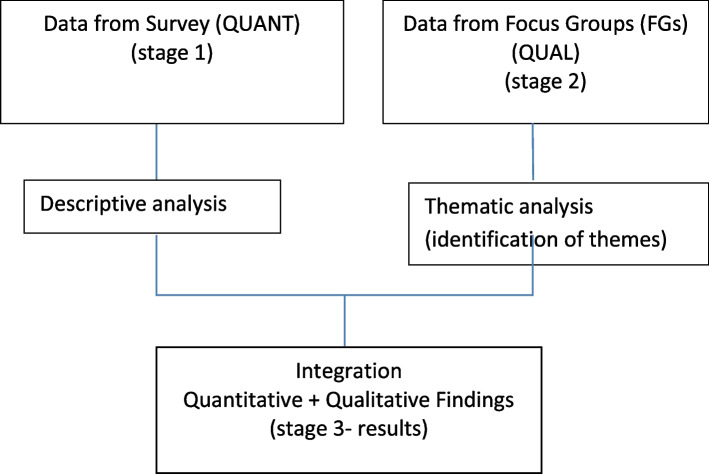
Flow chart of sequential mixed methods designs for study

Quantitative findings (from the survey) were compared with findings from the qualitative data (from the FGs). The data from both were analysed to see if they were consistent or inconsistent. The results were brought together using 'joint displays’ in the form of tables where quantitative and qualitative were viewed in parallel columns to identify similarities (consistencies) and differences (inconsistencies). Consistency and inconsistency were highlighted in the integration phase (third column on the joint display tables). The joint displays are a frequently used approach to provide a structure from which interpretation is transparent and meaningful [16, 17]. Integrated findings are discussed and compared to other published preceptorship data in the discussion. Figure 1 outlines the process of the mixed methods design.

## Results

### Demographics

**Fig. 2:**
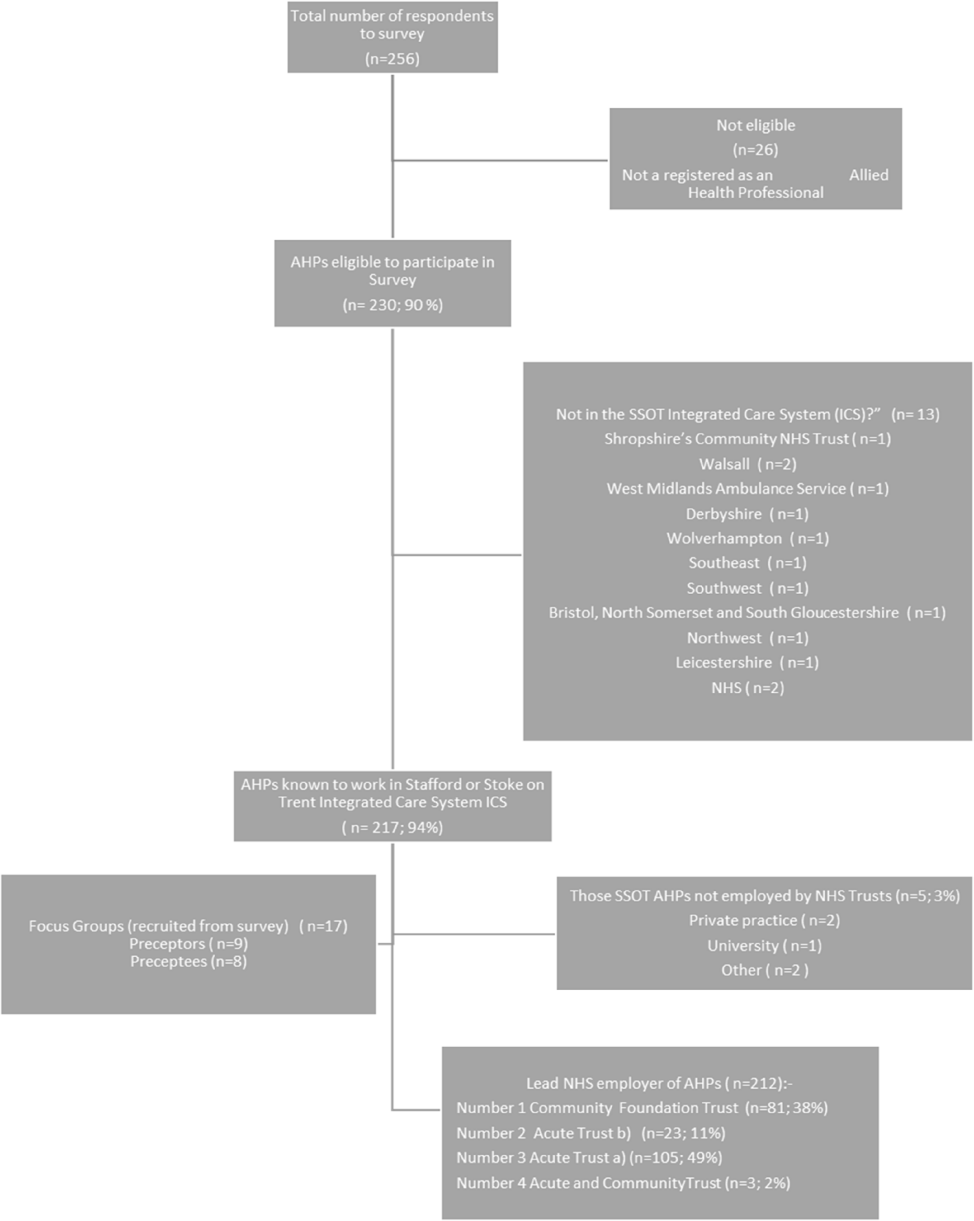
Flow Diagram of AHPs involved in the preceptorship study

Of 256 survey respondents, 230 were eligible as qualified UK AHPs (Fig. 2); 94% (*n* = 217) being from SSOT. This represents approximately 10% of the estimated AHP workforce of the SSOT ICS. Respondents from all four NHS Provider Trusts in SSOT were represented in the survey and FGs. Organisation number 1 was over-represented in both the survey (*n* = 81/217; 38%) and focus groups (*n* = 9/17; 53%). Only 3% of all respondents (*n* = 5/217) were non- NHS employees, although the number of AHPs working in private, independent or voluntary organisations in SSOT is not known. Only AHPs employed by the NHS articipated in the FGs.

Demographics of respondents are illustrated in Table 2. The majority of respondents, 79% (*n* = 181) were female. Sixty (26%) of respondents identified themselves as preceptees and 109 (47%) as preceptors, 17% were neither, but identified as being service managers or practice educators for AHP students. Data of survey respondents reflected a workforce between the age of 21 and over 55. Not unexpectedly, the majority of preceptees, 75% (*n* = 45) were under the age of 30 years, whereas preceptors were older, 77% (*n* = 84) over the age of 30.

**Table 2:** Demographic data of survey respondents

Demographic domain	All respondents (*n* = 230) (including Managers)	Newly qualified preceptees (*n* = 60)	Preceptors (*n* = 109)
**Gender**	n (%)	n (%)	n (%)
Male	32 (14)	11 (18)	16 (15)
Female	181 (79)	46 (77)	85 (78)
Not stated	17 (7)	3 (5)	8 (7)
**Age**			
21–25	38 (16)	28 (47)	9 (8)
26–30	43 (19)	17 (28)	16 (15)
31–35	34 (15)	6 (10)	19 (17)
36–45	58 (25)	4 (7)	32 (30)
46–55	37 (16)	3 (5)	22 (20)
55 +	18 (8)	2 (3)	11 (10)
Not stated	2 (1)	0 (0)	0 (0)

The ethnicity of respondents reflects the local population with the predominance of “White British” category (*n* = 206/230; 89%) followed by Indian/ Pakistani/other Asian. The ethnicity of non-white respondents at 9% does not reflect the 23% BAME workforce of the English National Health Service in the West Midlands [1].

**Fig. 3:**
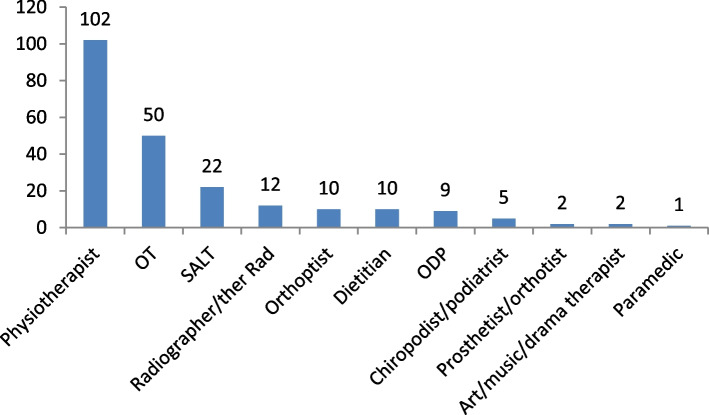
Survey respondents by AHP profession (*n* = 230) Key: OT = Occupational Therapist; ODP = Operating Department Practitioner; SALT = Speech & Language Therapist; Ther Rad = Therapeutic Radiographer

Thirteen of the 14 AHP professions responded to the survey (only Osteopathy not represented) and eight AHP professions were represented in the FGs. Physiotherapists comprised 44% of survey respondents (*n* = 102/230) and 24% of FG participants (*n* = 4/17). A breakdown of respondents of the survey by AHP profession is detailed in Fig. 3.

Results from the survey questionnaire revealed that 38% of all 217 respondents (*n* = 83) reported that their employing organisation had a preceptorship programme; of these 5 described them as being “service-specific”, 12 as “AHP-specific”, 23 as “profession specific” and 26 as Trust-specific. Others were unable to say.

Following transcript scrutiny 28 codes, 10 subthemes and 5 themes were identified from the preceptee FG, and 56 codes, 10 subthemes and 5 themes were developed from the preceptor FG. Table 3 illustrates how the final three themes were identified from the focus groups.

**Table 3:** Derivation of sub-themes and themes identified from the combined focus group codes

Theme	Sub-theme	Example of preceptee codes	Example of preceptor codes
Provision and format of preceptorship	Logistics Flexible but specific format Supportive	Extend preceptorship length for COVID graduates Same profession preceptor favoured Want AHP targeted preceptorship Reassurance needed	Frequency of preceptor meetings Face to face versus virtual delivery Mental health risk of being overwhelmed Individualised support needed
The impact of COVID-19	Changed AHP practice Graduate vulnerability	Patient complexity Less exposure to variety of patients Restricted placements	Service disruption Graduate confidence Fear in workplace Student experience gap
Future preceptorship strategy	Senior staff “buy in” Valuing preceptorship	Desire to understand healthcare “big picture” Employer commitment required to protect staff time Named preceptor important Varied senior support	Future recommendations Preceptor education needed Confusing terminology Protected time

Preceptees reported two unique themes: perceived value and benefits of preceptorship, and bespoke AHP-tailored preceptorship.

Preceptors specifically reported concern about confusing terminology surrounding preceptorship, and made recommendations about future improvement of AHP preceptorship in SSOT ICS.

Three over-arching themes were identified. Two themes were developed from the combined survey questionnaire data and the focus group findings identified by both preceptees and preceptors. The third theme, however, was identified solely from the qualitative FG data.

The three themes were:

- 1. Provision and format of preceptorship delivery
- 2. Impact of COVID-19
- 3. Future AHP preceptorship strategy

#### The provision and format of preceptorship delivery

Survey results illustrated a variable provision of AHP preceptorship across the SSOT ICS, and a lack of awareness and understanding about preceptorship. 53% (*n* = 31) of preceptee and 58% (*n* = 36) of preceptor respondents, did not think they had a preceptorship programme in their organisation. Those that were aware that a preceptorship existed within their organisation, lacked clarity about what it involved and for whom it was provided. This was particularly strong in the preceptor group where one of the preceptors was under the impression that a preceptorship was for all new starters, not just for newly qualified professionals. The lack of awareness of the programmes was thought in part to be due to a lack of familiarity with preceptorship terminology. Preceptor participants reported confusion around the term ‘preceptorship’ (a term often more associated with nursing colleagues) and were unclear how it differed from mentorship, induction and continuous professional development (CPD). It was clear that the lack of understanding around the terminology for both the preceptee and preceptors was a barrier to optimising existing preceptorship provision.

*“I’ve no idea what this is and I didn’t know whether to recommend them to go to it or not” [Preceptor SLT]*

*“I do not know what a preceptorship is, to be perfectly honest … no one has mentioned this before.” [Preceptee PT]*

The reported format of the preceptorship was variable: some provision was tailored specifically to AHPs, whilst others were organised by other professions and reported to be targeted towards nursing colleagues. This was negatively received by many FG participants:

*”[Preceptorship] needs to be profession specific within AHP umbrella … . They speak to a room of nurses, but it isn’t a room of nurses. They [the presenters] need to acknowledge individuals in the room as well, otherwise they switch off” [Preceptee OT].*

There was a consensus from both preceptors and preceptees across all AHP professional groups that an AHP or profession specific preceptorship would be of greater value than one that included other health professionals.

Typically respondents reported preceptorship lasting for the first year of newly qualified practitioners’ employment and were arranged monthly. There were inconsistencies about how AHP preceptorship was delivered. Apart from the radiography and podiatry professions, new graduates were not allocated a named mentor. Very few (8%; 5/60) of respondents reported receiving group preceptorship, the majority (52%; 33/63) of meetings were in person, face to face with a preceptor (71%; 45/63). A minority (24%; 15/63) of preceptees reported meetings were remotely delivered using virtual platforms. Peceptees across all professional groups greatly valued the face-to-face meetings and many wanted a named preceptor,

*“Knowing that I had a dedicated point of contact who was very experienced who made themselves available for any queries” [Preceptee OT].*

However the preceptor FG highlighted that 1:1 meetings placed demands on their workloads which was sometimes difficult to accommodate, especially if this hadn’t been built into their (the preceptors) job plans. This was acknowledged as a potential barrier to implementation. A preceptor highlighted that a potential solution to reduce some of the time commitments for the preceptors was to introduce support in the form of a 1:1 ‘buddy scheme’ whereby newly qualified AHP preceptees would be linked to other preceptees for peer and pastural support.

There was also terminology- confusion around how preceptorships differed from CPD and supervision, with preceptors being the most unclear, by frequently using the terms interchangeably. Generally, across both FGs and across the professions, it was agreed that there is benefit to group preceptorship for AHP specific issues. In particular preceptor participants expressed enthusiasm for this focusing on three of the four pillars of learning: facilitated learning, leadership and research, which in part, could be held online; whereas the face to face 1:1 sessions would focus more on the profession specific clinical practice element. A few preceptees expressed concern that education around research and development could be overwhelming for some if placed at the beginning, and recommended that instead these components would be best placed towards the end of the preceptorship. To enable optimal timing, some proposed a flexible “rolling programme” of preceptorship that covered core agreed generic AHP offers could enable preceptee timely choice, a suggestion broadly welcomed by both preceptors and preceptee participants.

There was a lack of information on how effective the current preceptorship programmes were, particularly from the preceptors. This could be due to the lack of awareness of preceptorship programs, the lack of feedback from organisations to AHP preceptors about the effectiveness of the programs, or the preceptees not sharing their views of the preceptorship programs with the preceptors. By not providing this feedback, preceptors are less likely to engage with the preceptorship program (See Table 4).

**Table 4:** Provision and format of SSOT AHP preceptorship: joint displays with mixed methods interpretation

Quantitative findings (stage 1)	Qualitative findings (stage 2)	Mixed method integration (stage 3)
**Definition & awareness of preceptorship**		
53% (*n* = 31/59) of preceptee respondents reported being unsure/did not think they had a preceptorship programme 57% (*n* = 36/63) of preceptor respondents reported there was no current preceptorship programme or they lacked awareness of one	Respondents were asked if they had a Preceptorship Programme *“I do not know what a preceptorship is, to be perfectly honest … no one has mentioned this before.” [preceptee PT]* *“I feel more awareness around what the preceptorship is/does would be extremely useful.” [preceptee SLT]* *“A clear preceptorship programme would have been very helpful when newly qualified” [preceptee OT]* *“I’ve no idea what this is and I didn’t know whether to recommend them* (the preceptees*) to go to it or not” [preceptor SLT]* *“First I heard about it* (preceptorship) *was when two of my staff got invited to it” [preceptor OT]* *“Our preceptorship programme is designed to be new to post, so that’s all levels. We haven’t got a band 8 one yet ‘[Managerial and advanced practitioner level] but we’re working on it” [preceptor radiographer]* *“I sort of see the preceptorship as the bit at the start of the NHS journey” [preceptor PT]*	Consistent lack of awareness for preceptorship programmes not helped by a lack of clarity around the definition or for whom it is for
**Format of preceptorship (tailored to AHPs)**		
Of the 38% (*n* = 83/217) reporting they had a preceptorship programme in their organisation, 28% (*n* = 23/83) described it being “profession-specific”, 31% (*n* = 26/83) Trust specific, 14% (*n* = 12/83) AHP specific, 6% (*n* = 5/83) service specific. 15% (*n* = 13/83) were unable to say	*“Preceptorship should not just be focussed on nursing but the other AHP's roles that are out there. [preceptee ODP]* *”[Preceptorship] Needs to be profession specific within AHP umbrella …. They speak to a room of nurses, but it isn’t a room of nurses. They [the presenters] need to acknowledge* *individuals in the room as well otherwise they switch off” [preceptee OT]* *“[the Preceptorship] Programme has been vague, a minimal presence of AHPs … I am still not fully* *aware of the nursing role in my team as [I’ve] been working from home/ remotely, and [this] has* *impacted on areas of my role which has been incredibly challenging …” [preceptee OT]*	Consistency that few preceptorship programmes are AHP/profession specific
**Delivery of preceptorship**		
52% (*n* = 33/63) of meetings were offered on 1:1 basis. The majority (71%, *n* = 45/63) were face to face. 24% (*n* = 15/63) of preceptorships were delivered via a remote platform or by phone. Only 8% (*n* = 5/60) of respondents reported receiving group preceptorship The majority of preceptees (67%, *n* = 74/111) reported not being allocated a named preceptor. The lack of a named preceptor was widespread across most AHP professions (except for Radiography and Chiropody). Although numbers are small, this appears to be a particular concern for Paramedics (*n* = 1/1; 100%), ODPs (*n* = 2/3; 66%), Orthoptists ((*n* = 1/3; 33%) and Physiotherapists (*n* = 9/27; 33%) Survey results identified that preceptorship meetings with preceptors were offered to preceptees most frequently on a monthly basis (*n* = 14/60; 23%), although 10% (*n* = 6/60) reported being offered weekly meetings 57% (*n* = 32/56) of respondents reported sessions lasting between 30 min and 2 h with both preceptee and preceptor devising the content (*n* = 36/53; 68%) Most preceptorships lasted one year in duration (*n* = 46/54; 85%)	*“Knowing that I had a dedicated point of contact who was very experienced who made themselves available for any queries. Working with different clinicians with different clinical specialisms to gain a more rounded experience.” [preceptee OT]* *“Preceptorship training should be more widely available for staff members to understand and give 1–1 support to newly qualified members of staff.” [preceptor ODP]*	Inconsistency around how the preceptorship was being delivered e.g. face-to- face or digital; group or 1:1 meetings There was consistency that most preceptorships lasted for one year
**Perceived Value & Effectiveness of preceptorship**		
Many preceptees (*n* = 23/54; 43%) reported the preceptorship programmes to be very effective, another 42% (*n* = 23/54) suggested they were somewhat effective, but 15% (*n* = 8/54) described them being ineffective Of the 41% of preceptors (*n* = 19/46) who knew of a preceptorship programme for newly qualified AHPs in their organisation, 85% (*n* = 33/39) declined to comment when asked to grade the effectiveness of any known preceptorship programme in their organisation, possibly reflecting a reluctance to comment or little information known about preceptorship arrangements for newly qualified staff	*“One [rotation] where I didn’t meet anyone until four months in, you’re assuming you’re working fine as no one has told you otherwise ….. you’re never really told what you are doing is right, until you sit down and have that conversation” [preceptee PT]*	There were mixed views on how effective the current preceptorship programmes were

Key: *ODP* Operating Department Practitioner, *OT* Occupational Therapist, *PT* Physiotherapist, *SLT* Speech and Language Therapist

#### Impact of COVID-19 pandemic on AHPs

A synthesis of the data aimed to understand the impact of the COVID-19 pandemic on AHPs. There was doubt whether existing AHP preceptorship offers were still fit for purpose.

There were consistent findings that due to altered exposure AHP students had received during their placements over the pandemic, ill equipped them to working in real life clinical environments as newly qualified practitioners. The preceptors strongly expressed concerns that newly qualified AHPs were stressedby their new professional roles, a view substantiated by respondent preceptees. 30% (24/82) confessed they had considered leaving their first job and 12% (10/82) had admitted to feeling “overwhelmed” in the survey.

Preceptors reported little, if anything, had changed in their preceptorship offer since COVID and that the current preceptorship schemes had not adapted sufficiently to address the new needs of graduates. Only 5% (4/82) preceptee respondents believed that their preceptorship offer had changed as a result of COVID.

*“I had a mixture of shadowing in clinics above my competency … A mishmash because of COVID, it’s been really, really, difficult” [Preceptee podiatrist].*

20% (16/82) of preceptee respondents admitted to always or frequently having insufficient work support, resulting in additional anxiety about lack of support when working remotely, which seemed to affect some professions more than others.

“*I am still not fully aware of the nursing role in my team as [I’ve] been working from home / remotely and [this] has impacted on areas of my role which has been incredibly challenging … ” [Preceptee OT].*

Preceptees working in smaller professional groups or teams found the lack of clinical experience as a student was compounded by working in isolated environments such as in the community setting as a newly qualified practitioner.

*“I have had to repeatedly remind people that I am newly qualified. There has been an assumption of knowledge and competency. I have [had] to push to seek that support rather than it being readily available” [Preceptee OT]*

Fears around newly qualified AHP staff retention directly because of COVID were emotively conveyed by some preceptors.

*“We have got a lot of very challenging situations from graduates who may/ may not want to take up a career, [but have] gaps in knowledge … they are such a fragile section of the workforce ” [Preceptor PT].*

Another expressed an urgency to address this:

*“In another 12 months, if they can’t cope with these pressures, we will lose them” [Preceptor PT]*

Other preceptors reported anxiety in final year students lacking confidence:

“*A lot of students are coming to me now saying’ I am not ready to qualify’ … [Preceptor Podiatrist].*

One operating department practitioner (ODP) commented:

“*Our service has gone straight through the roof to try and clear a back log … and these newly qualified staff are coming into this when we are at a peak [with patient caseloads], with less staff and more work … . and they will be filling the gaps with less support” [Preceptor ODP ].*

Many preceptees were also aware of this, some recommending a more gradual exposure to caseload pressures as a counter strategy.

Additional file 1 illustrates how this second theme was derived from survey data and focus group discussions.

#### Future strategy for preceptorship

The majority of respondents valued preceptorship. 43% (23/54) of preceptees believing what they had received to be effective, although 42% (23/54) had described it as ‘somewhat effective’ and 15% (8/54) as ‘ineffective’. The value of support was expressed by one new graduate participant:

*“There was one [rotation] where I didn’t meet anyone until four months in … you’re assuming you’re working fine as no one has told you otherwise … .. but you’re never really told what you are doing is right, until you sit down and have that conversation” [Preceptee PT].*

Preceptors were less certain about effectiveness; 85% (33/39) of survey respondents declined to rate the preceptorship offer in their organisation. Only 41% (19/46) of respondents knew if such an offer existed. In the focus group discussion, preceptors were broadly very supportive of preceptorship for newly qualified staff in particular. This theme led to discussion about suitability of their existing provision and possible future improvements.

The future strategy for preceptorship became a large discussion topic, particularly for the preceptor FG. There was unanimous agreement across the different AHP professions and across different AHP providers (community/ acute hospital based etc.) that some key factors influenced the effectiveness of existing AHP preceptorship.

Firstly, preceptors recognised that a key barrier for them was insufficient time allocated for being a preceptor. Preceptees also remarked that the challenges of outpatient/community waiting lists and ward pressures meant prioritising their own development was difficult.

Potential enablers discussed included building dedicated and protected time into job plans for both receiving and providing preceptorship across all grades of staff.

*“Throughout all bandings we need the right amount of preceptorship support/ supervision that they require put into their job plans, & the supervisor needs that time set aside as well- it’s incredibly important otherwise we have the risk of losing loads of people from our professions” (Preceptor Speech and Language Therapist).*

*“It is massively important that job planning and any preceptorship programme supports the preceptors, releasing them.. giving them time … they are still trying to balance that with their clinical workload” [Preceptor PT].*

Preceptors were clear that organisations need to understand the value of preceptorships for AHPs.

*“We need complete and utter “buy in” [from senior AHP and Trust management] … I know a couple of soon to be qualified (ODPs) who have said that they may want to go to other Trusts as the preceptorship that they offer is more robust that what we have currently got” [Preceptor ODP].*

Preceptees reported a perception that a number of organisations valued nursing preceptorships, but were less supportive of AHP preceptorship.

“*Preceptorship should not just be focussed on nursing but the other AHP's roles that are out there” [Preceptee ODP]*

Preceptors highlighted that organisations should work collaboratively to ensure that preceptorship is provided equally and fairly across organisations and to look at means to provide shared programs to avoid unnecessary duplication and cost or burden, although differences between AHP professions were reported.

*“Trusts need to work closely with professional bodies with regards to preceptorship to avoid duplication. My professional body requires newly qualified practitioners to complete their preceptorship to be able to gain full registration, which is already a lot of work”[Preceptor Speech and Language Therapist].*

Others actively wanted better profession-specific alignment and even guidance from professional bodies at a national level.

*“A more set and organised preceptorship program would help to standardise knowledge and skill set among diagnostic radiographers, ensuring that one radiographer has very similar skill sets compared to others, even if working in different hospitals … and preceptorship programmes would be especially valuable for diagnostic radiographers working in specialist modalities, as a lot of this education comes on the job as opposed to being taught at university” [Preceptor Radiographer]*

Many, but not all participants reiterated how important they perceived preceptorship to be, even prioritising it over patient care.

*“it's so incredibly important to get it right … As Ops lead and Team leads we have to put in that challenge and say ‘ NO’ [i.e. prioritise preceptorship] sometimes” [Preceptor Speech and Language Therapist].*

Several participants suggested AHP managers should integrate time for preceptorship in job plans for both preceptees and preceptors as well as ensuring organisations design and provide robust and AHP-focused preceptorship programmes that align to professional body requirements and enable consistent provision across a system.

**Fig. 4:**
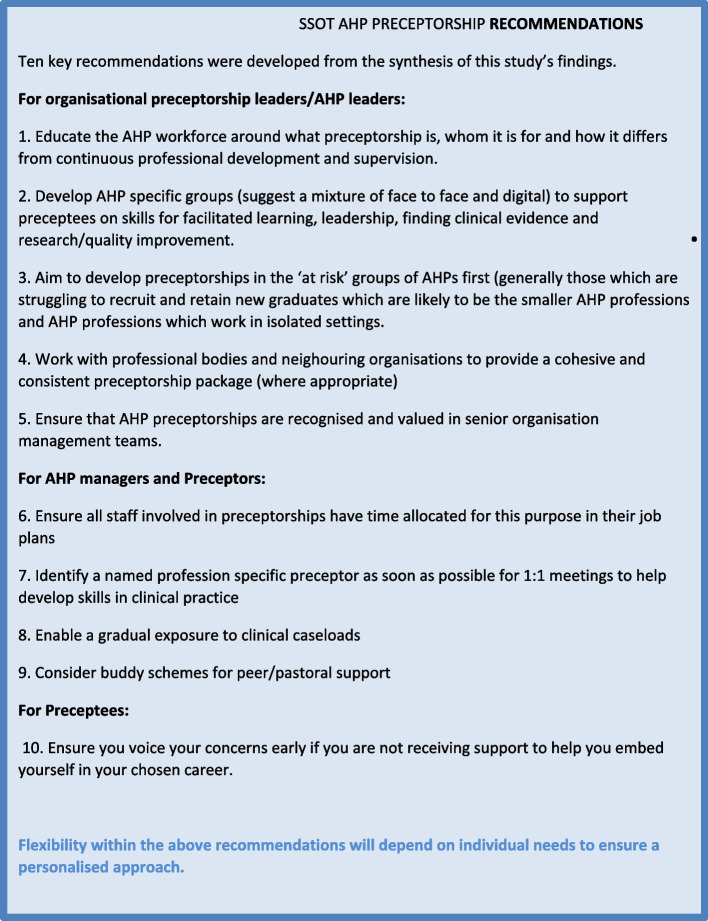
Key study recommendations

It was evident from both survey results and focus group findings that there was a lack of knowledge and understanding about preceptorship in SSOT. Substantial AHP preceptorship discrepancies were identified between SSOT healthcare providers, although a clear consensus about its value for newly qualified staff, especially for graduates who had been negatively impacted by COVID. Proposals for improvements to existing provision were enthusiastically discussed. Recommendations (See Fig. 4) were developed from the findings of this study.

## Discussion

Participants from all AHP disciplines (except osteopathy) in the SSOT ICS (*n* = 230) responded to a survey questionnaire and 17 consented and participated in subsequent focus groups. Across the ICS preceptorship provision reported inconsistent findings in content, delivery and perceived effectiveness. There were consistent findings especially from focus group participants who concluded that Covid-19 had led to newly qualified AHP practitioners being less well prepared for their graduate healthcare roles and that the preceptorship programmes, when available, were not adequately supporting them. Preceptors raised concerns regarding potential junior staff retention issues if preceptorship programmes were not fully embedded into practice. This study benefitted from representation of 13 out of 14 AHP professions working within the SSOT ICS.

Comparisons to AHP and nursing preceptorship studies reveal similar findings relating to building professional confidence and reducing stress [18–20], the need to improve access to programmes [18], a need for a cultural/organisational level of support for preceptorship programmes [18], the need for profession specific good preceptor:preceptee relationship with 1:1 sessions [18–22] in combination with group preceptorship programs to provide a multi-strategy approach [23], better peer support systems (such as ‘buddies’) [22] and aligns with reports that preceptors need adequate support themselves [19, 22, 24]. One paper on AHPs (therapeutic radiographers) reported consistent findings to this study in that undergraduate training during COVID-19 amplified the anxieties and challenges faced by newly qualified professionals [25]. In addition, nursing preceptors have also reported concerns about staff attrition rates in the absence of effective preceptorship programmes [20, 21, 26] and have also highlighted a need for preceptorship programmes to evolve in light of enforced changes in service delivery as a consequence of the pandemic [27]. There is limited and inconsistent reports on whether Continuous Professional Development (CPD) generally, or preceptorship specifically affects rate of attrition, despite that fact that this has been widely recognised as a key reason to support the provision of a preceptorship [3]. A review published on AHPs (occupational therapists) reported a lack of correlation between CPD and attrition [28]. Further studies are required to identify whether attrition rates reduce for AHPs receiving a preceptorship. A theme identified from one healthcare review, but not mirrored in the findings of our study, was that group preceptorship programmes had a greater impact on individuals, compared to those using a one-to-one preceptorship model [29]. One preceptorship study on AHPs (occupational therapists) recommended that staff in the preceptorship year should not rotate posts until the preceptorship program is complete [23]. This last theme was not something identified as an issue in this study.

Comparing our study findings to the recommendations from England’s Department of Health, Framework (2010) [3] and the Health Education England Standards (2015) [4], we found that none of the recommendations had been fully met across all organisations. Some recommendations had been integrated in some Trusts (e.g. the provision of an organisation wide lead, protected learning time for preceptorship) but in many cases, there was no evidence that recommendations had been implemented in any way (e.g. preceptorships are monitored and evaluated to demonstrate value for money). This study provides a clear indication that much work needs to be done to meet the current national recommendations.

The project team planned a determined strategy to obtain as wider professional representation from SSOT AHPs as possible and this was largely successful. Eleven of the of the 14 AHP professions (as defined by NHS England), were represented in the survey. All four NHS Trust providers engaged with the project.

The study was not without limitations. An over-representation of some professions, and under representation of others, was observed when comparing survey respondents with proportion of current AHP English Health and Care Professions Council registrants. There was also relatively limited engagement (2% of respondents) from the 4th provider Trust, however this was a small organisation with fewer AHPs. Survey respondents were predominantly from a white ethnic background (90%) and only one ethnic minority was represented in the focus groups. The lack of representation of AHPs from the minority groups and those working in the private, independent and voluntary sector was a limitation of this study. In addition, The SSOT AHP Faculty were keen to generate meaningful recommendations before the 2021 “COVID graduates” started employment, and as such, there was no time to recruit “matched” preceptor and preceptee participants on aspects such as gender, ethnicity and profession etc. in the focus groups. It is acknowledged that although it would have been interesting to compare findings from matched characteristics for the preceptee and preceptor perspectives, the time constraints were prohibitive. Although this was a small study we had representation from the large majority of AHP professions, meaning that some aspects are likely to be generalizable given that SSOT is not atypical to other UK Integrated Care Systems.

For reasons which are unclear, AHP Preceptorship is largely under-reported. Our study highlights strong beliefs that AHP preceptorship is vital, but also identifies significant concerns, about efficacy and equity of provision across different AHP professions working in different organisations across an ICS. A ‘perfect storm’ scenario of newly qualified graduate AHP staff, with varying digital competence, who have had less undergraduate clinical exposure due to COVID and consequently lack confidence, are entering a pressured health service, with a backlog of caseloads from the start of their career.

Future research exploring the impact of a generic AHP preceptorship “package” for SSOT AHP staff is planned. Planned SSOT evaluations will measure preceptee confidence, AHP staff recruitment and retention across all provider organisations, with a view to improving the health and wellbeing and job satisfaction of AHP staff, which will ultimately then be able to offer better patient care in the post pandemic SSOT healthcare economy. Additional evaluation of changes made to SSOT preceptorship is scheduled.

Preceptorship for AHPs across the UK is evolving but is only recently being explored. It is our intention and hope therefore, that by sharing these evidence-based pragmatic AHP preceptorship recommendations, which we believe to be transferable across the UK, newly qualified staff will be able to access strategically timed flexible support and guidance, relevant to their specific needs, during their first year in post. Benefits to AHP staff as well as their patients are anticipated and clearly align with England’s NHS “People Plan” (2020).

### Supplementary Information

**Additional file 1.** The Impact of COVID-19pandemic on preceptorships- Joint Displays with Mixed Methods Interpretation.

## Data Availability

The datasets used and/or analysed during the current study available from the corresponding author on reasonable request.
