# Supplementary material for: Evaluation of Staffordshire, Stoke on Trent Allied Health Professionals preceptorship programmes: a mixed method UK study
Source: BMC Med Educ. 2023 Aug 21;23:591. doi: 10.1186/s12909-023-04515-7 (PMC10441690; doi:10.1186/s12909-023-04515-7)
Supplement: Supplementary file 1 — Additional file 1. The Impact of COVID-19pandemic on preceptorships- Joint Displays with Mixed Methods Interpretation. [file 12909_2023_4515_MOESM1_ESM.docx]

**Additional file 1: The Impact of COVID-19 pandemic on preceptorships- Joint Displays with Mixed Methods Interpretation**

| **Quantitative Findings (stage 1)** | **Qualitative Findings (stage 2)** | **Mixed Method Integration (stage 3)** |
| --- | --- | --- |
| **AHP role changes**  Many preceptees reported being either always or frequently being stressed (n=7/82; 9%) or overwhelmed (n=10/82; 12%).  Preceptees reported to always or frequently  have concerns over insufficient work support (n=16/82; 20%) and workload concerns (n=10/82;12%).  30% (n=24/82) of the preceptees stated they had considered leaving their roles | Both preceptor and preceptee groups emphasised that the newly qualified AHP roles had changed dramatically due to COVID, potentially leading to higher rates of attrition of this workforce in the future and suggesting therefore the importance of preceptorship is essential now.  *“I have had to repeatedly remind people that I am newly qualified. There has been an assumption of knowledge and competency. I have [had] to push to seek that support rather than it being readily available” [Preceptee OT]*  Preceptors expressed concerns about newly qualified AHPs not having been exposed to a clinical working culture and much less hands on clinical experience in the same way students before them had  *“(Because of covid) we have got a lot of very challenging situations from graduates who may/ may not want to take up a career, [but have] gaps in knowledge…they are such a fragile section of the workforce ” [preceptor PT]*  “ *A lot of students are coming to me now saying’ I am not ready to qualify’…[Preceptor Podiatrist ]*  *Our service has gone straight through the roof to try and clear a back log and these newly qualified staff are coming into this when we are at a peak [with patient caseloads], with less staff and more work…. and they [new graduates] will be filling the gaps with less support” [preceptor ODP ]*  *“In another 12 months, if they can’t cope with these pressures, we will lose them”) [preceptor PT]* | Consistency that many newly qualified AHPs are at risk of leaving their professions due to feeling stressed, overwhelmed, underprepared and under supported. |
| **Changes to Delivery of preceptorship because of COVID-19**  Of the preceptees who thought their preceptorship programme had been adapted due to COVID, 71 % (n=15/21) reported the biggest change to be the method of delivery (from face to face to on-line). Only 5% reported any change in content of preceptorship programme as a result of COVID.  Just over half of the preceptees (n=22/43; 51%;) had no awareness of, or didn’t know if any adaptations had been made to their preceptor programme due to COVID. | Preceptees voiced concerns regarding not being fully equipped to take on a qualified role.  *“A preceptorship would bring someone from degree and settle them (newly qualified) into routine work, [but] this routine work wasn’t there… I had a mixture of shadowing in clinics above my competency… A mishmash because of covid, it’s been really really difficult” [preceptee podiatrist]* | Consistent evidence that existing/current preceptorship programmes are not providing sufficient support to newly qualified AHPs who had completed much of their undergraduate training during the COVID-19 pandemic. |
